# Effect of small peptide chelated iron on growth performance, immunity and intestinal health in weaned pigs

**DOI:** 10.1186/s40813-023-00327-9

**Published:** 2023-07-07

**Authors:** Limei M. Sun, Bing Yu, Yuheng H. Luo, Ping Zheng, Zhiqing Huang, Jie Yu, Xiangbing Mao, Hui Yan, Junqiu Luo, Jun He

**Affiliations:** 1 Institute of Animal Nutrition, Sichuan Agricultural University, Chengdu, 611130 Sichuan Province P. R. China; 2 Key Laboratory of Animal Disease-resistant Nutrition, Chengdu, 611130 Sichuan Province P. R. China

**Keywords:** Small peptide chelated iron, Production performance, Immune capacity, Intestinal health, Weaned piglets

## Abstract

**Background:**

Small peptide chelated iron (SPCI), a novel iron supplementation in pig diets, owns growth-enhancing characteristics. Although a number of researches have been performed, there is no clear-cut evidence to show the exact relationship between the dose and effects of small peptide chelated minerals. Therefore, we investigated the effect of dietary supplementation of SPCI at different doses in the growth performance, immunity, and intestinal health in weaned pigs.

**Methods:**

Thirty weaned pigs were randomly assigned into five groups and feed with basal diet or the basal diet containing 50, 75, 100, or 125 mg/kg Fe as SPCI diets. The experiment lasted for 21 d and on day 22, blood samples were collected 1 h later. The tissue and intestinal mucosa samples were collected following.

**Results:**

Our results showed that the feed to gain ratio (F:G) decreased with different levels of SPCI addition (*P* < 0.05). The average daily gain (ADG) (*P *< 0.05) and digestibility of crude protein (*P* < 0.01) decreased with 125 mg/kg SPCI addition. With dietary different levels of SPCI addition, the serum concentrations of ferritin (quadratic, *P* < 0.001), transferrin (quadratic, *P* < 0.001), iron content in liver (quadratic, *P* < 0.05), gallbladder (quadratic, *P* < 0.01) and fecal (quadratic, *P* < 0.01) increased quadraticly. While the iron content in tibia (*P* < 0.01) increased by 100 mg/kg SPCI supplementation. Dietary 75 mg/kg SPCI addition increased the serum insulin-like growth factor I (IGF-I) (*P* < 0.01) and SPCI (75 ~ 100 mg/kg) addition also increased the serum content of IgA (*P* < 0.01). The serum concentrations of IgG (quadratic, *P* < 0.05) and IgM (quadratic, *P* < 0.01) increased quadraticly by different levels of SPCI supplementation. Moreover, different levels of SPCI supplementation decreased the serum concentration of D-lactic acid (*P* < 0.01). The serum glutathione peroxidase (GSH-Px) (*P* < 0.01) elevated but the malondialdehyde (MDA) (*P* < 0.05) decreased by 100 mg/kg SPCI addition. Interestingly, SPCI supplementation at 75 ~ 100 mg/kg improved the intestinal morphology and barrier function, as suggested by enhanced villus height (*P *< 0.01) and villus height/crypt depth (V/C) (*P* < 0.01) in duodenum, as well as jejunum epithelium tight-junction protein ZO-1 (*P* < 0.01). Moreover, SPCI supplementation at 75 ~ 100 mg/kg increased the activity of duodenal lactase (*P* < 0.01), jejunal sucrase (*P* < 0.01) and ileal maltase (*P* < 0.01). Importantly, the expression levels of divalent metal transporter-1(*DMT1*) decreased with different levels of SPCI addition (*P* < 0.01). In addition, dietary SPCI supplementation at 75 mg/kg elevated the expression levels of critical functional genes such as peptide transporter-1(*PePT1*) (*P* = 0.06) and zinc transporter 1 (*ZnT1*) (*P* < 0.01) in ileum. The expression levels of sodium/glucose co-transporter-1 (*SGLT1*) in ileum (quadratic, *P *< 0.05) increased quadraticly by different levels of SPCI addition and amino acid transporter-1 (*CAT1*) in jejunum(*P* < 0.05) also increased by 100 mg/kg SPCI addition.

**Conclusions:**

Dietary SPCI supplementation at 75 ~ 100 mg/kg improved growth performance by elevated immunity and intestinal health.

## Background

Iron (Fe) is an indispensable trace element for animals, which was responsible for various biological events such as hematopoiesis, oxygen transport, DNA synthesis and energy metabolism [1, 2]. Moreover, Fe plays critical roles in the differentiation of adaptive immune cells (e.g. T lymphocytes) and maturation of the systematic immunity [3–5]. Weaned piglets are in a rapid growth phase and need a lot of iron to meet the needs of increasing blood volume and number of red blood cunts (RBCs) [6]. Piglets can easily become iron deficient because of low body iron stores and increased iron requirements [6]. Previous studies indicated that iron deficiency in pigs not only induce anemia or related dysfunctions, but can also appear severe oxidative stress, which impairs the intestinal barrier functions by inducing apoptosis of the intestinal epithelial cells [7–9].

Intestinal iron absorption from dietary is an important way to meet the needs of increasing blood volume and number of RBCs [10]. Therefore, iron supplementation in diets is the common strategy to prevent weaned piglets from iron deficiency. Previous study also indicated that diets supplemented with 150 mg of Fe (Fe sulfate) per kilogram of diet may be necessary to maintain blood profiles in pigs [11, 12]. Inorganic irons such as the ferrous sulfate and ferrous fumarate are the most popular iron supplements as they are really accessible and commercially feasible[13]. In recent year, organic irons such as the glycine and methionine chelated irons have attracted considerable research interest, as they are more palatable and effective than the inorganic irons [13, 14]. The SPCI is a novel organic iron, which has been looked as the fourth generation of the iron supplements. As compared to other forms of iron, the SPCI have a couple of advantages such as an elevated chemical stability, a higher absorption rate, and a higher biological titer [15].

Currently, various small peptide chelated trace elements have been widely used in animal nutrition and feed industry. For instance, dietary supplementation of the small peptide chelated trace minerals was found to improve the growth performance and antioxidant capacity in broilers [16]. Similarly, dietary supplementation of SPCI at low doses (300 and 500 mg/kg) significantly improved the antioxidant capacity in broiler chickens [17]. Although a number of researches have been performed, there is no clear-cut evidence to show the exact relationship between the dose and effects of mall peptide chelated minerals, as their effects on animals are closely related to the animal species, sex, and the physiological stage of animals. Previous reported that dietary iron supplementation from 0 to 120 mg/kg linearly increased ADG in weaned piglets [18]. Therefore, we selected the four doses (50,75,100 and 125) for gradient addition to preliminarily explore the dose of the new iron supplement SPCI in weaned piglets and to investigate the effect of dietary supplementation of SPCI at different doses on growth performance, immunity, and intestinal barrier functions in weaned pigs.

## Materials and methods

The animal experiment in this study was carried out after approved by the Animal Care and Use Committee of Sichuan Agricultural University (Chengdu, China, No.20,210,111). The SPCI (Fe ≥ 15.0%; chelate ratio ≥ 90%; MW of the small peptide: 180–500 Da) was kindly provided by Shuxing Biotechnology Co., Ltd (Jiangsu, China).

### Animals and management

Thirty weaned boars from the same littermates (Large White × Landrace × Duroc) with an average body weight (9.91 ± 0.10 kg) were randomly assigned into five groups, each treatment with six pigs, and fed with basal diet (T1) or SPCI diets containing 50, 75, 100, and 125 mg/kg Fe (T1 ~ T5). All piglets were vaccinated against pseudorabies and swine fever.

The experiment lasted for 21d and the basal diet ( Table S1) was formulated to meet the nutrient requirements recommended by the National Research Council 2012 [19]. The feed is fed in the form of powder. The 0.7 m × 1.5 m metabolic cages placed in the heated room (25–28 °C) were used to house pigs individually and each pig was given *ad libitum.* The room relatively humidity controlled at 55 ~ 65%.

### Growth performance

The average daily gain (ADG) was calculated according to the BW of each pig measured On day 1 and 22 after 12 h fasting. The feed intake of each pig was recorded daily to determine the average daily feed intake (ADFI), and the G:F was calculated according to ADG and ADFI (F/G equals to ADFI dividing ADG).

### Sample collection and treatment

On d 18, we conducted four-day fresh fecal samples collection. On d 22, the bloods were obtained after 12 h fasting to obtain serum samples. The whole blood samples were used for the hematological analysis. Pigs were euthanized by intravenous sodium pentobarbital injection and then slaughtered by an exletting protocol. Then the tissue samples (liver, gallbladder, kidney, tibia) were obtained following and stored at − 80 °C immediately. Take approximately 4 cm of each duodenum, jejunum, and ileum tissues to fix in 4% paraformaldehyde solution for morphological analyses and immunofluorescence. Then Rinse central portion of duodenum, jejunum, and ileum tissues with 0.9% cold physiological saline and then the mucosa samples were scraped from duodenum, jejunum, and ileum segments.

### Apparent total tract nutrient digestibility analysis

The processed feed and fecal samples were used to measure the digestibility of nutrients. Use acid insoluble ash (AIA) as endogenous indicators, a method described by the Chinese National Standard (GB/T23742-2009). The content of dry matter (DM), crude protein (CP), ether extract (EE) and ash were measured according to AOAC, whereas the the gross energy (GE) content was determined by an adiabatic bomb calorimeter (LECO, St. Joseph, Michigan, USA). All contents were calculated by following formula: (100-A1F2/A2F1 × 100) [20]. A1: digesta nutrient; A2: digesta AIA; F1: diet AIA; F2: digesta AIA.

### Iron content in tibia and tissue samples

The tissue samples (liver, gallbladder, and kidney) and tibia were dried at 100 °C for 24 h and ashed at 550 °C for 10 h or 36 h respectively. The pre-treated samples were finally used to determine iron content according to the method of Shelton and Southern [21]. The nitric acid-perchloric acid mixture (1:1) were used to dissolve the ashed samples and then the distilled deionized water were used for dilution and the flame atomic absorption spectrophotometry (AA-6300, Shimadzu Corp., Tokyo, Japan) were used for analysis of iron [22].

### Serum parameter analysis

The Enzyme-linked kits (Jiangsu, China) can provide reliable indexes for the content determination of serum ferritin, transferrin, insulin-like growth factor-1, immunoglobulin subsets (IgG, IgM, IgA), D-lactate and diamine oxidase. All procedures correspond to the manual of the kits, respectively.

Catalase (CAT), malondialdehyde (MDA), glitathione peroxidase (GSH-PX), total superoxide dismutase (T-SOD), total antioxidant capacity (T-AOC) and urea nitrogen (BUN) in serum were determined using the commercial kits (Nanjing Jiancheng Biotechnology Co., Ltd, Nanjing, China).

### Intestinal morphology analysis

The paraffin-embedded duodenum, jejunum, and ileum samples were cut and then stained with hematoxylin and eosin (H&E) for the intestinal morphology examination under the Eclipse CI-L photo microscope (Nikon, Japan). The height of 5 intact villi and crypt depths were recorded by image-Pro Plus 6.0 analysis software.

### Immunofluorescence analysis

The immunofluorescence can provide reliable indexes for the detection of the ZO-1 protein distribution in jejunal tissues. The flushed samples were incubated with 1 mol/L ethylene diamine tetraacetic acid (EDTA, pH 9.0, Gooddbio Technology Co., Ltd., Wuhan, China) for antigen retrieval. Following this, the tissue sections were block with 3% bovine serum albumin and then incubate with rabbit anti-ZO-1 polyclonal antibody all night at 4 °C. All procedures correspond to our previous research [23].

### Flow cytometry assays

The proportion of jejunal apoptotic cells were determined by Flow cytometry and all procedures were conducted according to our previous report [23]. Briefly, isolate jejunal mucosal layer, followed by grinding and filtering to form a cell suspension. After that, take 100 µL of the cell suspension and put it into 5 mL tubes, and then add 5 µL PE Annexin V and 7-AAD in the tubes for incubation in the dark. Lastly, take Annexin V binding buffer (400 µL) to the reaction tubes then mixed it by a vortex. The jejunal apoptotic cells were detected by CytoFLEX flow cytometry (Beckman Coulter, Brea, CA, USA) within 1 h.

### Enzyme activity

The commercial kits (Nanjing Jiancheng Biotechnology Co., Ltd. Nanjing, China) were used to detect the digestive enzymes in duodenal, jejunal, and ileal mucosa including lactase, sucrase, and maltase.

### RNA isolation, reverse transcription, and real-time quantitative PCR

Divalent metal transporter-1 (DMT1), peptide transporter-1 (PePT1), cationic amino acid transporter-1 (CAT1), zinc transporter-1 (ZnT1), Na+-dependent glucose transporter-1 (SGLT1), and glucose transporter-2 (GLUT2) mRNA levels of intestinal mucosa were analyze by quantitative real-time PCR and the experimental procedure refer to the method described by Wan et al. [24]. The primer sequences synthesized commercially by Shenggong Bioengineering (Shanghai, China) were presented in Table S2.

### Statistical analysis

The data were analysed by single factor variance of SPSS 24.0 (SPSS, Inc). The Duncan method was used for multiple comparisons, and the treatment effects of different iron addition gradients were linear and quadratic Regression analysis. *P* < 0.05 and 0.05 < *P* < 0.10 was considered significant and as trend respectively when compare the differences between the CON group and the SPCI groups.

## Results

### Effect of SPCI on growth performance and nutrients digestibility in weaned pigs

The body weight was observed on the 21th day of SPCI supplementation (Table 1). The average daily feed intake (ADFI) and feed to gain ratio (F:G) decreased with dietary different levels SPCI addition (*P* < 0.05). The average daily gain (ADG) decreased with 125 mg/kg SPCI addition (*P* < 0.05). There were no significant differences in the digestibilities of dry matter (DM), gross energy (GE), and ether extract (EE), but the apparent digestibility of crude protein (CP) decreased in T5 group (*P* < 0.01).

### Effect of SPCI on iron transportation and deposition in weaned pigs

With dietary SPCI addition, the serum concentrations of ferritin and transferrin increased quadraticly (quadratic, *P* < 0.01) and the best performance was observed in T3 group (Table 2). Moreover, the iron content in liver (quadratic, *P* < 0.05), gall bladder (quadratic, *P* < 0.01) and fecal (quadratic, *P* < 0.01) increased quadraticly by dietary SPCI addition. Meanwhile, the iron content in the tibia (*P* < 0.01) increased by SPCI supplementation at a higher dose (100 mg/kg).

**Table 1:** Effect of SPCI on growth performance and nutrients digestibility in weaned pigs^1^

ITEM^2^			Treatments^3^				SEM	*P*-value		
	T1	T2		T3	T4	T5		ANOVA	Linear	Quadratic
IBW (kg)	9.75	9.88		9.83	9.92	10.18	0.10	0.73	0.24	0.42
FBW (kg)	14.53	14.64		14.59	14.71	14.19	0.12	0.10	0.63	0.50
ADG (g/day)	227.62^a^	226.67^a^		226.59^a^	228.41^a^	190.56^b^	10.20	< 0.05	0.03	< 0.01
ADFI (g/day)	481.33^a^	418.06^b^		417.62^b^	409.98^b^	365.40^b^	4.06	0.04	< 0.01	< 0.01
F:G	2.12^a^	1.84^b^		1.85^b^	1.79^b^	1.94^ab^	0.04	0.02	0.02	< 0.01
DM (%)	89.18	90.13		90.02	88.83	88.56	0.76	0.15	0.46	< 0.05
CP (%)	81.92^a^	84.87^a^		82.88^a^	83.03^a^	79.03^b^	0.56	< 0.01	0.75	0.06
EE (%)	79.21	77.66		82.30	80.74	80.60	0.32	0.48	0.21	0.47
GE (%)	87.74	88.68		88.89	87.42	87.98	0.25	0.49	0.53	0.07

^1^Mean and total SEM are list in Separate columns (n = 6)

^2^IBW, initial body weight; ADG, average daily gain; ADFI,average daily feed intake; F:G,feed to gain ratio; DM, dry matter; CP, crude protein; EE, ether extract

^3^T1, control (basal diet); T2, the basal diet containing 50 mg/kg SPCI ; T3, the basal diet containing 75 mg/kg SPCI ; T4, the basal diet containing 100 mg/kg SPCI; T5, the basal diet containing 125 mg/kg SPCI.

^a,b^ Means in the same row with no common superscripts differ significantly (P < 0.05)

**Table 2:** Effect of SPCI on iron transportation and deposition in weaned pigs^1^

ITEM			Treatments^2^				SEM	*P*-value		
	T1	T2		T3	T4	T5		ANOVA	Linear	quadratic
Ferritin (ng/mL)	122.03^c^	233.28^ab^		264.35^a^	188.38^b^	187.06^b^	11.42	< 0.01	0.14	0.00
Transferrin (µg/mL)	16.48^c^	23.22^b^		31.97^a^	23.07^b^	21.74^b^	1.06	< 0.01	0.09	0.00
Liver (mg/kg)	30.37^b^	42.80^ab^		40.56^ab^	57.04^a^	48.86^ab^	3.23	0.08	0.01	0.04
Gall bladder (mg/kg)	6.52^b^	10.38^a^		11.30^a^	10.58^a^	11.50^a^	0.59	0.04	0.01	0.01
Kidney (mg/kg)	16.53	19.62		18.55	20.50	22.50	1.00	0.44	0.13	0.32
Tibia (mg/kg)	197.83^b^	155.33^b^		182.33^b^	256.83^a^	187.83^b^	9.62	< 0.01	0.24	0.50
Fecal (mg/kg)	651.99^d^	1040.89^c^		1007.39^c^	1231.39^b^	1401.95^a^	58.38	< 0.01	< 0.01	< 0.01

^1^Mean and total SEM are list in Separate columns (n = 6)

^2^T1, control (basal diet); T2, the basal diet containing 50 mg/kg SPCI ; T3, the basal diet containing 75 mg/kg SPCI ; T4, the basal diet containing 100 mg/kg SPCI; T5, the basal diet containing 125 mg/kg SPCI.

^a-c^ Means in the same row with no common superscripts differ significantly (P < 0.05)

### Effect of SPCI on serum parameter in weaned pigs

Serum parameter analysis revealed that dietary SPCI addition at 75 mg/kg increased the serum concentration of IGF-I ( (Table 3, *P* < 0.01) and the concentration of IgA was elevated by SPCI at a dose of 75 and 100 mg/kg (*P* < 0.01). Meanwhile, the serum concentrations of IgG (quadratic, *P* < 0.05) and IgM (quadratic, *P* < 0.01) increased quadraticly by SPCI supplementation. Interestingly, different levels of SPCI supplementation decreased the serum concentration of D-lactic acid (*P* < 0.01). Moreover, SPCI supplementation at 100 mg/kg decreased the serum concentration of MDA (*P* < 0.05) and increased the serum concentration of GSH-Px (*P* < 0.01).

**Table 3:** Effect of SPCI on serum parameter in weaned pigs^1^

ITEM^2^			Treatments^3^				SEM	*P*-value		
	T1	T2		T3	T4	T5		ANOVA	Linear	quadratic
IGF-1 (µg/L)	35.31^bc^	36.34^b^		41.22^a^	32.21^c^	32.14^c^	0.82	< 0.01	0.27	0.01
IgA (µg/mL)	20.20^b^	19.41^b^		38.16^a^	32.83^a^	22.09^b^	1.97	< 0.01	0.27	0.42
IgG (µg/mL)	51.15^c^	245.30^a^		163.47^b^	134.01^b^	113.68^b^	15.28	< 0.01	0.10	0.02
IgM (µg/mL)	19.05^c^	37.12^a^		31.34^b^	26.92^b^	28.31^b^	1.28	< 0.01	0.36	< 0.01
DAO (pg/mL)	226.83^ab^	190.02^b^		254.41^a^	241.58^ab^	206.55^ab^	8.22	0.07	0.96	0.99
D-lactate (µg/L)	468.53^a^	316.06^b^		300.62^b^	289.39^b^	332.90^b^	14.02	< 0.01	< 0.01	< 0.01
BUN (mmol/L)	6.21	5.56		5.51	5.48	5.58	0.17	0.66	0.19	0.24
GSH-Px (µmol/L)	241.26^b^	255.21^b^		217.45^b^	310.50^a^	242.18^b^	9.01	< 0.01	0.84	0.98
T-AOC (U/mL)	0.49^ab^	0.49^ab^		0.62^ab^	0.67^a^	0.44^b^	0.03	0.09	0.41	0.53
MDA (nmol/mL)	3.09^a^	2.87^a^		2.23^ab^	1.94^b^	2.24^ab^	0.14	0.03	0.15	0.19
CAT (U/mL)	21.90	30.84		26.73	32.46	26.16	1.44	0.14	< 0.05	0.14
T-SOD(U/mL)	53.18	57.70		56.62	59.24	53.84	1.28	0.54	0.45	0.41

^1^Mean and total SEM are list in Separate columns (n = 6)

^2^IGF-I, insulim-like growth factor 1; IgA, immunoglobulins A; IgG, immunoglobulins G; IgM, immunoglobulins M; DAO, diamine oxidase; BUN, blood urea nitrogen; GSH-Px, Glutathione peroxidase; T-AOC, total antioxidant capacity, MDA, Malondialdehyde; CAT, Catalase; T-SOD, total superoxide dismutase

^3^T1, control (basal diet); T2, the basal diet containing 50 mg/kg SPCI ; T3, the basal diet containing 75 mg/kg SPCI ; T4, the basal diet containing 100 mg/kg SPCI; T5, the basal diet containing 125 mg/kg SPCI.

^a-c^ Means in the same row with no common superscripts differ significantly (P < 0.05)

### Effect of SPCI on intestinal morphology and mucosal enzyme activity in weaned pigs

**Fig. 1:**
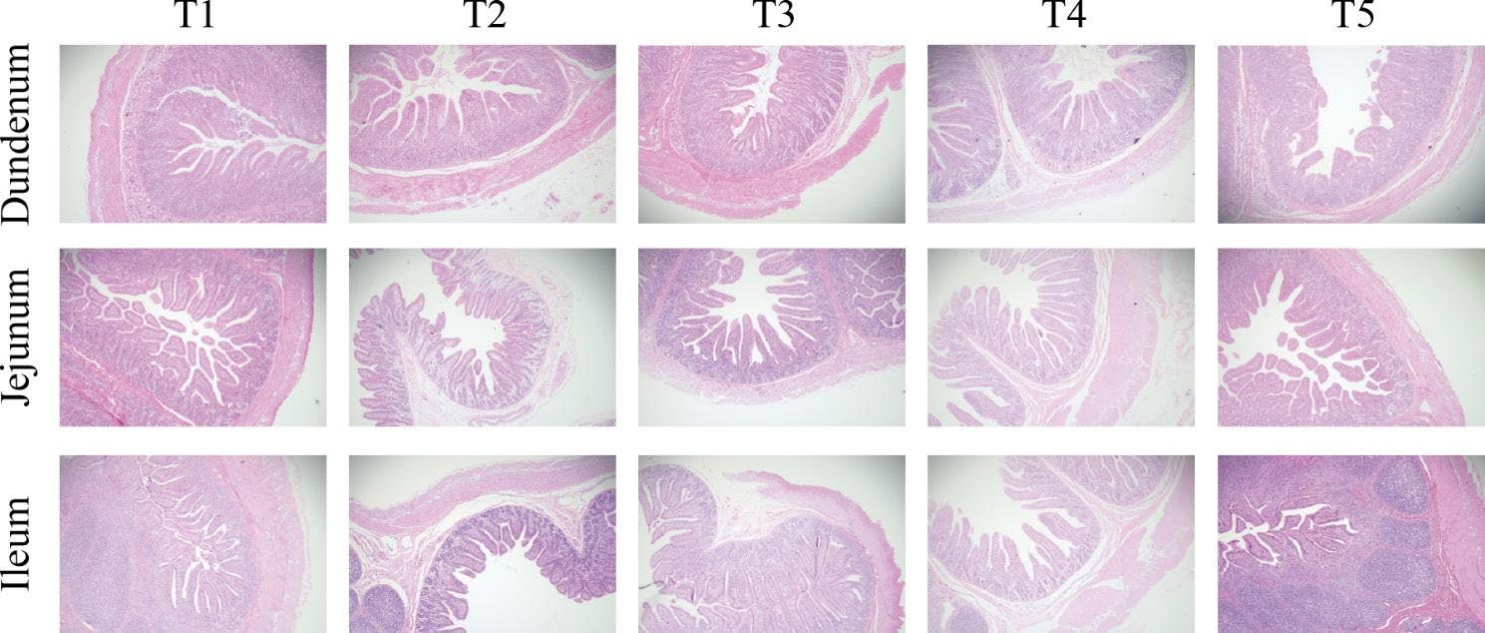
**Effect of SPCI on intestinal morphology in weaned pigs (H&E; × 40)** T1, control (basal diet); T2, the basal diet containing 50 mg/kg SPCI ; T3, the basal diet containing 75 mg/kg SPCI ; T4, the basal diet containing 100 mg/kg SPCI; T5, the basal diet containing 125 mg/kg SPCI.

Intestinal morphologic and biometric analysis revealed that SPCI supplementation at 75 ~ 100 mg/kg elevated the villus height (*P* < 0.01) and the ratio of villus height to crypt depth (V/C) (*P* < 0.01) in the duodenum (Table 4; Fig. 1 ). Meanwhile, SPCI supplementation at 75 mg/kg elevated the villus height (*P* < 0.05) in the ileum. The intestinal mucosal enzyme activity analysis (Table 5) indicated that dietary SPCI supplementation at 75 ~ 100 mg/kg significantly increased the activities of duodenal lactase (*P* < 0.01), jejunal sucrase (*P *< 0.01) and ileal maltase (*P *< 0.01). Meanwhile, SPCI added at 75 mg/kg also increased the activities of duodenal maltase (*P *< 0.01) and jejunal lactase (P < 0.01). Moreover, the activities of duodenal sucrase (*P* < 0.01) also increased by SPCI added at a higher dose (100 mg/kg).

**Table 4:** Effect of SPCI on intestinal morphology in weaned pigs^1^

ITEM^2^			Treatments^3^				SEM	*P*-value		
	T1	T2		T3	T4	T5		ANOVA	Linear	quadratic
duodenum										
Villus height (µm)	355.51^c^	425.15^bc^		520.29^a^	449.22^b^	395.70^bc^	14.23	< 0.01	0.11	< 0.01
Crypt depth (µm)	258.95^a^	168.69^bc^		137.72^c^	142.01^c^	211.18^ab^	11.34	< 0.01	0.02	< 0.01
V/C	1.42^d^	2.62^bc^		3.84^a^	3.30^ab^	1.95^ cd^	0.20	< 0.01	0.06	< 0.01
Jejunum										
Villus height (µm)	370.08^ab^	361.39^ab^		408.94^a^	324.69^b^	299.49^b^	12.28	0.04	0.07	0.02
Crypt depth (µm)	216.83	178.61		191.62	199.47	191.50	8.40	0.72	0.38	0.65
V/C	1.78	2.10		2.07	1.71	1.62	0.11	0.54	0.60	0.22
Ileum										
Villus height (µm)	319.55^c^	322.73^ab^		371.70^a^	332.19^ab^	292.28^c^	13.23	0.03	0.67	0.03
Crypt depth (µm)	182.16	170.27		159.42	172.73	163.44	13.74	0.76	0.25	0.50
V/C	1.77	2.00		2.38	1.96	1.82	0.79	0.10	0.56	0.09

^1^Mean and total SEM are list in Separate columns (n = 6)

^2^ V/C, Villus height: Crypt depth

^3^T1, control (basal diet); T2, the basal diet containing 50 mg/kg SPCI ; T3, the basal diet containing 75 mg/kg SPCI ; T4, the basal diet containing 100 mg/kg SPCI; T5, the basal diet containing 125 mg/kg SPCI.

^a-c^ Means in the same row with no common superscripts differ significantly (P < 0.05)

**Table 5:** Effect of SPCI on digestive enzyme activities in the intestinal mucosa (U/mgprot)^1^

ITEM			Treatments^2^				SEM	*P*-value		
	T1	T2		T3	T4	T5		ANOVA	Linear	quadratic
Duodenum										
Lactase	2.53^c^	2.56^c^		7.18^a^	4.72^b^	3.84^bc^	0.36	< 0.01	0.06	0.14
Sucrase	10.38^b^	12.32^ab^		13.11^ab^	16.02^a^	6.14^c^	0.86	< 0.01	0.10	< 0.01
Maltase	12.57^b^	15.14^b^		19.95^a^	16.41^ab^	14.14^b^	0.69	< 0.01	0.18	0.18
Jejunum										
Lactase	40.41^b^	32.92^bc^		63.10^a^	30.67^bc^	22.50^c^	3.22	< 0.01	0.23	0.34
Sucrase	6.01^b^	5.50^b^		15.83^a^	14.45^a^	5.90^b^	1.17	< 0.01	0.29	0.45
Maltase	36.63^ab^	30.40^ab^		49.91^a^	32.77^b^	23.50^b^	2.68	0.02	0.39	0.70
Ileum										
Lactase	1.13^ab^	1.69^a^		1.62^a^	0.77^b^	1.12^ab^	0.11	0.02	0.80	0.77
Sucrase	24.77	19.88		31.52	22.40	18.59	2.00	0.32	0.61	0.61
Maltase	23.96^c^	65.76^a^		62.05^a^	41.48^b^	28.14^bc^	3.75	< 0.01	0.61	< 0.01

^1^Mean and total SEM are list in Separate columns (n = 6)

^2^T1, control (basal diet); T2, the basal diet containing 50 mg/kg SPCI ; T3, the basal diet containing 75 mg/kg SPCI ; T4, the basal diet containing 100 mg/kg SPCI; T5, the basal diet containing 125 mg/kg SPCI.

^a-c^ Means in the same row with no common superscripts differ significantly (P < 0.05)

### Effect of SPCI on distribution of tight-junction protein ZO-1and apoptosis in

#### Intestinal epithelial cells

**Fig. 2:**
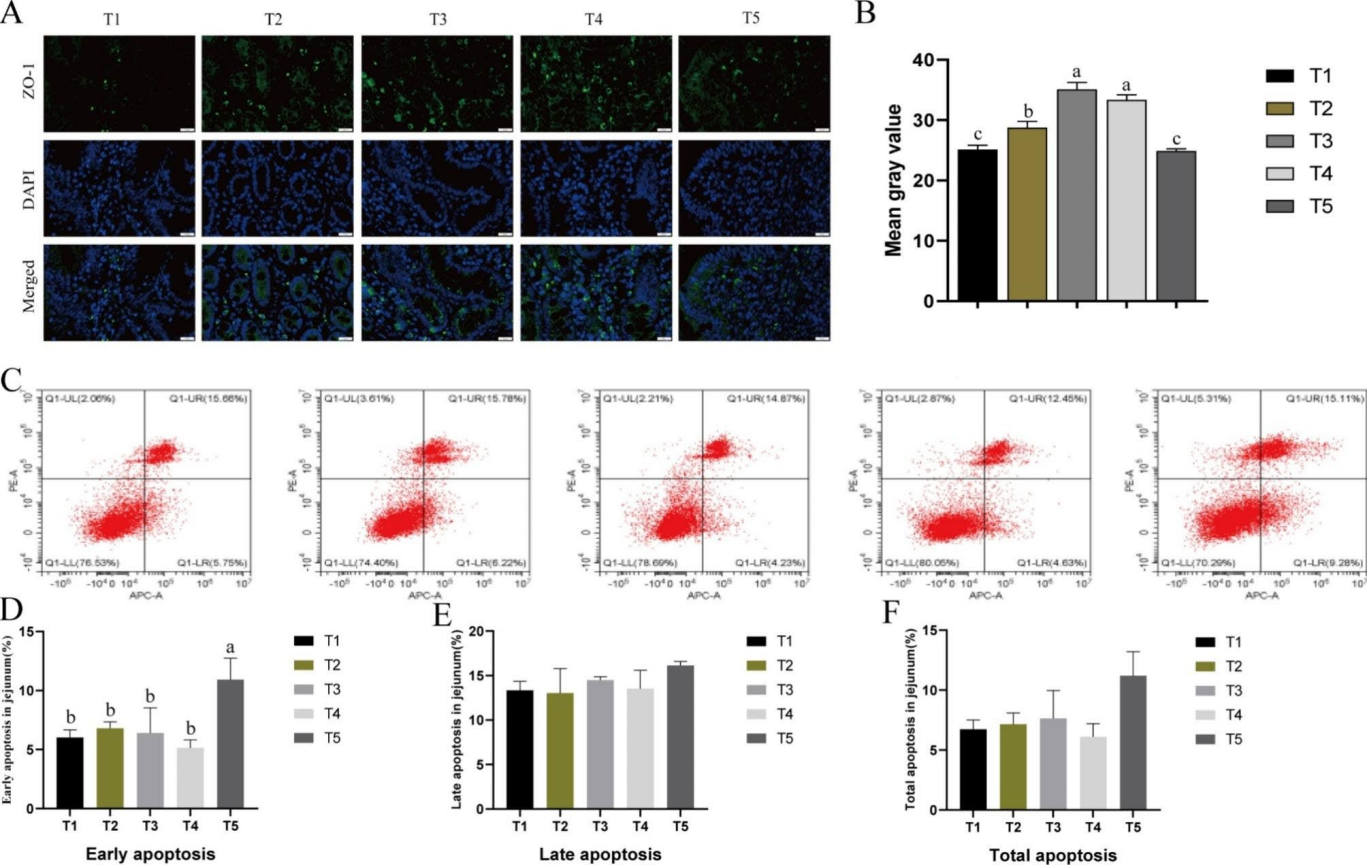
**Effect of SPCI on ZO-1 distribution and apoptosis of the intestinal epithelium cells** (**A**) Localization of ZO-1 and DAPI (DNA) in the jejunum. ZO-1 protein (green), DAPI stain (blue) as well as merged ZO-1 protein and DAPI are presented; (**B**) The mean gray value of ZO-1; (**C**) Evaluation of jejunal cell apoptosis by flow cytometry in weaned pigs. 30,000 cells were used in each acquisition reading. Frames were divided into 4 quadrants: Q1-UL represents necrotic cells; Q1-UR represents late apoptotic and early necrotic cells; Q1-LR represents early apoptotic cells; and Q1-LL represents normal cells; Percentages of apoptotic cells of early apoptosis (**D**), late apoptosis (**E**), total apoptosis (**F**) in the jejunum, respectively. a,b,c Mean values with different letters on vertical bars indicate significant differences (*P* < 0·05). T1, control (basal diet); T2, the basal diet containing 50 mg/kg SPCI ; T3, the basal diet containing 75 mg/kg SPCI ; T4, the basal diet containing 100 mg/kg SPCI; T5, the basal diet containing 125 mg/kg SPCI

The abundance of tight-junction protein ZO-1 in the jejunum epithelium (*P* < 0.01) increased with dietary SPCI addition at a dose from 50 to 100 mg/kg (Fig. 2). Dietary SPCI supplementation at a higher dose ( 125 mg/kg) increased the ratio of early apoptosis cells (*P* < 0.01), but had no significant influence on the ratio of total and late apoptosis cells (*P* > 0.05).

### Effect of SPCI on the expression levels of critical genes related to nutrient digestion and absorption

**Fig. 3:**
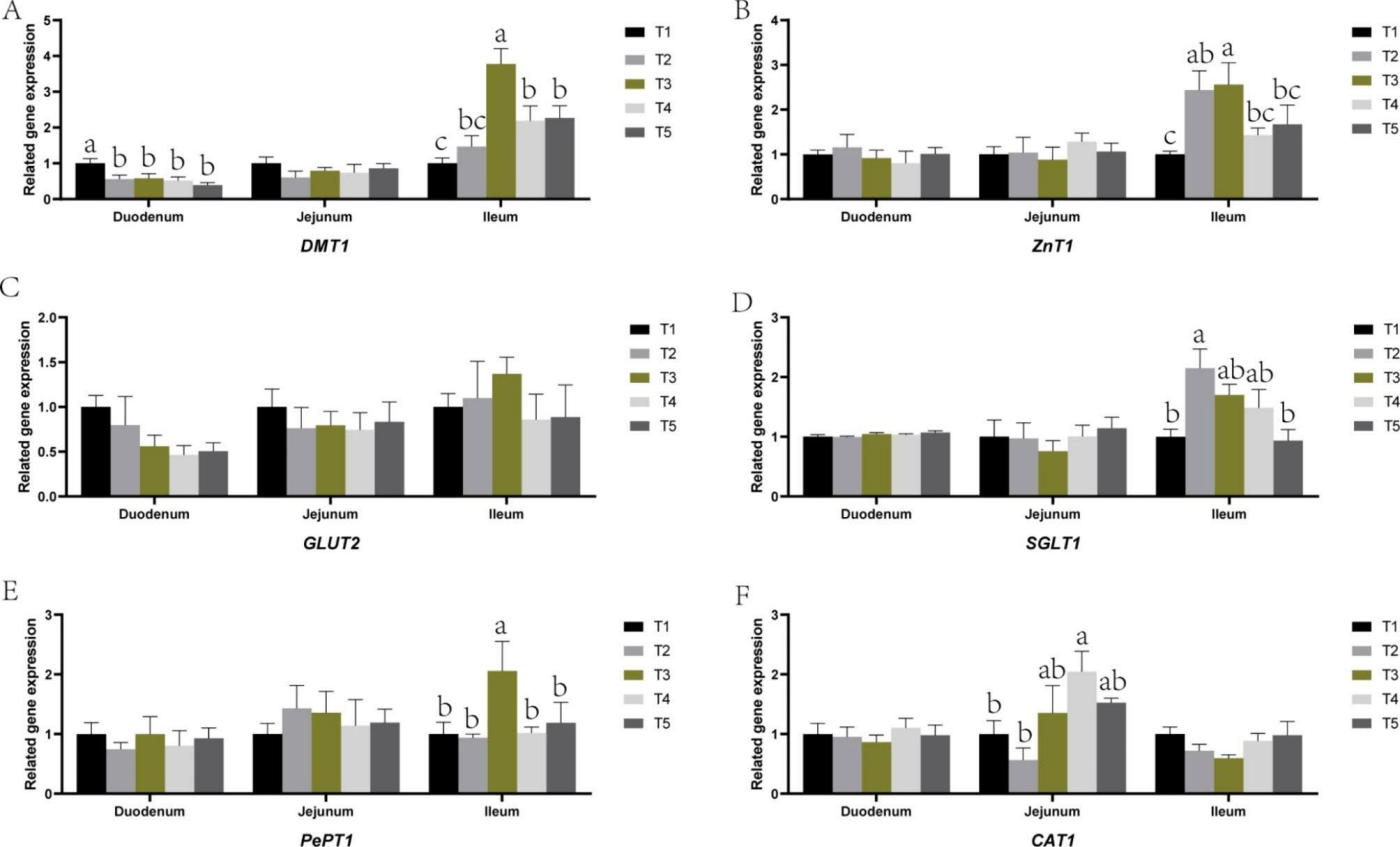
**Effect of SPCI on the expressions levels of critical genes related to nutrient digestion and absorption** DMT1, divalent metal transporter-1(A); ZnT1, zinc transporter-1(B); GLUT2, glucose transporter-2(C); SGLT1, sodium glucose transport protein-1(D); PePT1, peptide transporter-1(E); CAT1, amino acid transporter-1(F); a,b,c Mean values with different letters on vertical bars indicate significant differences (*P* < 0.05). T1, control (basal diet); T2, the basal diet containing 50 mg/kg SPCI ; T3, the basal diet containing 75 mg/kg SPCI ; T4, the basal diet containing 100 mg/kg SPCI; T5, the basal diet containing 125 mg/kg SPCI

The expression levels of critical genes in Fig. 3 displayed that different levels SPCI supplementation decreased the expression levels of *DMT1* in ileum (*P* < 0.01) but the expression levels of *DMT1* in ileum increased (*P* < 0.01) by 75 ~ 100 mg/kg. The expression levels of *SGLT1* in ileum increased quadraticly (quadratic, *P* < 0.05) by SPCI supplementation. The expression levels of *ZnT1* (*P* < 0.01) and *PePT1* (*P *< 0.10) in the ileum mucosa elevated by SPCI supplementation at 100 mg/kg. Dietary SPCI supplementation at 100 mg/kg also elevated the expression level of *CAT1* in the jejunum mucosa (*P* < 0.05).

## Discussion

Iron is an essential trace element for animals including the pigs and it plays an important role in improving the growth performance and immunity [4, 5]. Previous study showed that as compared to other forms of iron, the SPCI has a higher absorption rate and a higher bioavialability [15]. In the present study, SPCI supplementation decreased the F:G value, indicating a positive effect on growth performance in the weaned pigs. The result is consistent with previous studies using different animal species [25–27].

Ferritin is the primary iron storage protein, which serve as a buffer to against iron deficiency or overload. Whereas, the transferrin is widely known as the main iron-containing protein, which has been implicated in regulating iron absorption, storage, and utilization [28]. Previous study indicated that transferrin is essential for cell growth and differentiation, and plays an important role in growth of neonatal pigs [29]. In the present study, the serum concentrations of ferritin and transferrin increased quadraticly by SPCI supplementation (P < 0.05). Meanwhile, dietary 100 mg/kg SPCI supplementation increased the iron deposition in tibia and tissues such as the liver, gall bladder, and kidney. Our results are similar to previous studies on pigs and the increased deposition of tissue iron may indicated an elevated absorption of iron from the gastrointestinal tract [27, 28]. Previous study indicated that iron deficiency inhibited the secretion of IGF-I and led to growth retardation in rats [30]. In this study, we found that SPCI supplementation at 75 mg/kg elevated the serum concentration of IGF-I. IGF-I regulates cellular metabolism and growth via endocrine, autocrine, and paracrine mechanisms [31]. These results may suggested that SPCI can promote animal growth by promoting IGF-1 secretion.

Iron is an essential component of many peroxide- or nitrous oxide-generating enzymes which is responsible for the function of the immune cells [32]. Immunoglobulins are a class of glycoproteins which were produced by plasma cells and play an important role in the immune response via recognizing and binding to particular antigens [33]. Immunoglobulins, according to their heavy chain subunits were classified into five categories containing IgA, IgD, IgE, IgG, and IgM [34]. The IgG as the main serum immunoglobulin among the five classes play a critical role in the phagocytosis of mononuclear macrophages and neutralisation the toxicity [35]. In our research, the serum concentrations of IgG and IgM increased quadraticly by SPCI supplementation and the concentration of IgA was elevated by SPCI at a dose of 75 and 100 mg/kg, which indicated an elevation of immunity in the weaned pigs. The results is similar to previous studies on pigs [36]. D-lactic is a class of toxic substances produced by gut microbes. And it was released into the circulation when the intestinal epithelial barrier is destroyed, leading to a significant increase in serum D-lactic level[37]. In our research, the serum D-lactic decreased quadraticly by SPCI supplementation, which suggested that SPCI can protect the intestinal structural integrity to some extent. Previous studies also indicated that dietary supplementation of organic irons can improve the antioxidant capacity in broilers [38, 39]. In this study, we found that SPCI supplementation at a higher dose (100 mg/kg) increased the serum concentration of GSH-Px and decreased the serum concentration of MDA. MDA, as a kind of lipid oxidation product, can reflect the degree of lipid peroxidation in the body [40]. Whereas, the GSH-Px can detoxify lipid peroxides and provide protection of cellular and subcellular membranes against ROS damage [3].

The digestion and absorption of nutrients were dependent on the integrity of intestinal epithelium [41, 42]. The villus height and crypt depth of the intestinal mucosa are the most commonly used indicators to evaluate the intestinal morphology [43]. In our research, dietary added SPCI with 75 ~ 100 mg/kg not only elevated the villus height and the ratio of V/C in duodenum, but also improved the villus height in ileum. Meanwhile, the villus height in jejunum increased quadraticly by SPCI supplementation . The abundance of tight-junction protein ZO-1 in the jejunum epithelium also increased with 75 ~ 100 mg/kg SPCI addition. ZO-1 was known as a crucial tight-junction proteins which directly or indirectly serve as the actin-based cytoskeleton and then forms a selective permeable barrier [44]. These results indicated an elevation of the intestinal epithelium integrity with SPCI addition. In our study, the number of early apoptotic cell in jejunum increased by SPCI supplementation at 125 mg/kg which is because excess iron can produce the reactive oxygen species (ROS) through the Fenton reaction, triggering intestinal cell apoptosis[45]. The enzymes present in small intestinal brush border membrane (lactase, sucrase, and maltase) were essential for mammalian animals including the pigs. The disaccharide enzymes, such as sucrase and lactase are involved in carbohydrate digestion and their activities will reduce during iron deficiency [46]. The activities of maltase and sucrase can provide as key markers to evaluate the development of intestinal [47, 48]. In the present study, SPCI supplementation at 75 ~ 100 mg/kg significantly increased the activities of duodenal lactase, jejunal sucrase and ileal maltase. The result is similar to previous studies on pigs [12, 49] and both results indicated an elevation on digestion and absorption of the intestinal epithelium by SPCI addition.

The *DMT1* found in the duodenal brush margin can mediate the entry of ferric ions into intestinal epithelial cells [50]. Studies have shown that the expression of *DMT1* will increase to promote iron absorption when the body is deficient in iron; conversely, when the body stores more iron, the expression of *DMT1* will decrease to inhibit iron absorption [51]. The expression levels of *DMT1* in duodenal mucosa was decreased upon different levels of SPCI addition in present study, which indicated that dietary SPCI supplementation can increase iron store in weaned pigs. Interestingly, the expression levels of *DMT1* in jejunal mucosa was increased by SPCI added at 75 ~ 100 mg/kg, which is related to the increased concentration of iron in the intestinal lumen because when the concentration of iron in the intestinal lumen increases, the expression of *DMT1* increases to ensure sufficient iron in intestinal villi cells [52]. The *PePT1* is known as one peptides transporters present in the intestinal epithelium, which is in charge of small peptides including dipeptides and tripeptides [53]. The *ZnT1* is the only zinc transporter predominantly located on the plasma membrane, where it plays a pivotal role exporting cytosolic zinc to the extracellular space [54]. In this study, SPCI supplementation at 75 mg/kg significantly elevated the expression levels of *PePT1* and *ZnT1* in the ileal mucosa. Cationic amino acid transporter 1 (*CAT-1*), which was act as the L-arginine transport carrier is related to NO generation, and because of this, it was also involved in regulating epithelial barrier function [55, 56]. Moreover, most of the absorption of cationic amino acids are depended on *CAT1* [57]. The expression levels of *CAT1* in jejunal mucosa was elevated upon SPCI added at 100 mg/kg in present study. The expression level of *SGLT1* in ileal mucosa was also increased quadraticly upon SPCI supplementation. The *SGLT1* is an active glucose transporter, which plays a critical role in maintaining glucose homeostasis at both physiological and pathological levels [58]. These results also indicated that dietary SPCI supplementation at a proper dose is beneficial for the intestinal barrier functions.

## Conclusions

In conclusion, our results indicated a beneficial effect of dietary SPCI supplementation at 75 ~ 100 mg/kg on the growth performance and intestinal health in the weaned pigs. The mechanisms behind its action might be associated with improved immunity and intestinal barrier functions. These features should make it an attractive iron supplement for use in animal nutrition and feed industry.

## Data Availability

All data generated or analysed during this study are available from the corresponding author on reasonable request.

## Abbreviations

ADFI: Average daily feed intake
ADG: Average daily body weight gain
AOC: Total antioxidant capacity
BUN: Blood urea nitrogen
CAT: Catalase
*CAT1*: Cationic amino acid transporter-1
CON: Control ( basal diet)
CP: Crude protein
DAO: Diamine oxidase
DM: Dry matter
*DMT1*: Divalent metal transporter-1
EE: Ether extract
FBW: Final body weight
G/F: The gain-to-feed ratio
GE: Gross energy
*GLUT2*: Glucosetransporter-2
GSH-Px: Glutathione peroxidase
IBW: Initial body weight
IgA: Immunoglobulins A
IGF-I: Immunoglobulins G
IgM: Immunoglobulins M
MDA: Malondialdehyde
*PePT1*: Peptide-transporter-1
*SGLT1*: Na+ -dependent glucose transporter-1
SPCI: Small peptide chelated iron
T-SOD: Total superoxide dismutase
V/C: Villus height Crypt depth
*ZnT1*: Zinc transporter-1
ZO-1: Zonula occludens
